# Longitudinal Genomic Evolution of Conventional Papillary Thyroid Cancer With Brain Metastasis

**DOI:** 10.3389/fonc.2021.620924

**Published:** 2021-06-23

**Authors:** Han Luo, Xue Liao, Yun Qin, Qianqian Hou, Zhinan Xue, Yang Liu, Feiyang Shen, Yuelan Wang, Yong Jiang, Linlin Song, Haining Chen, Lingyun Zhang, Tao Wei, Lunzhi Dai, Li Yang, Wei Zhang, Zhihui Li, Heng Xu, Jingqiang Zhu, Yang Shu

**Affiliations:** ^1^ Department of Thyroid and Parathyroid Surgery, National Clinical Research Center for Geriatrics, West China Hospital, Sichuan University, Chengdu, China; ^2^ State Key Laboratory of Biotherapy and Cancer Center, West China Hospital, Sichuan University, Chengdu, China; ^3^ Department of Radiology, West China Hospital, Sichuan University, Chengdu, China; ^4^ West China School of Medicine, Sichuan University, Chengdu, China; ^5^ Department of Pathology, West China Hospital, Sichuan University, Chengdu, China; ^6^ Department of Gastrointestinal Surgery, West China Hospital, Sichuan University, Chengdu, China; ^7^ Department of Clinical Pharmacology, Hunan Key Laboratory of Pharmacogenetics, Xiangya Hospital, Central South University, Changsha, China; ^8^ Department of Laboratory Medicine, State Key Laboratory of Biotherapy, West China Hospital, Sichuan University, Chengdu, China

**Keywords:** brain metastasis, thyroid cancer, cancer evolution, genetics, *CDK4*

## Abstract

**Background:**

Brain metastasis is extremely rare but predicts dismal prognosis in papillary thyroid cancer (PTC). Dynamic evaluation of stepwise metastatic lesions was barely conducted to identify the longitudinal genomic evolution of brain metastasis in PTC.

**Method:**

Chronologically resected specimen was analyzed by whole exome sequencing, including four metastatic lymph nodes (lyn 1–4) and brain metastasis lesion (BM). Phylogenetic tree was reconstructed to infer the metastatic pattern and the potential functional mutations.

**Results:**

Contrasting with lyn1, ipsilateral metastatic lesions (lyn2–4 and BM) with shared biallelic mutations of *TSC2* indicated different genetic originations from multifocal tumors. Lyn 3/4, particularly lyn4 exhibited high genetic similarity with BM. Besides the similar mutational compositions and signatures, shared functional mutations (CDK4*^R24C^*, TP53^R342*^) were observed in lyn3/4 and BM. Frequencies of these mutations gradually increase along with the metastasis progression. Consistently, *TP53* knockout and CDK4*^R24C^* introduction in PTC cells significantly decreased radioiodine uptake and increased metastatic ability.

**Conclusion:**

Genomic mutations in *CDK4* and *TP53* during the tumor evolution may contribute to the lymph node and brain metastasis of PTC.

## Introduction

Papillary thyroid cancer (PTC) comprises approximately 85% of thyroid cancer, which is the most common endocrine malignancy (1). According to the clinical reports, most of PTC patients experience favorable prognosis after surgery and appropriate adjuvant therapy, with >90% of 10-year overall survival (2, 3). Particularly, conventional papillary thyroid cancer (cPTC) is the most common PTC, and patients with cPTC experience even better treatment outcomes and lower relapse risk than other types of PTC (3). However, dedifferentiation is one of the most challenging entities for thyroid cancer, which induces high risk of distant metastasis and subsequent poor overall survival (4, 5). Brain metastasis (BM) is rarely observed, accounting for only 0.1–5% of all types of distant metastasis but predicting bleak prognosis with mean survival 7.1–33 months (6, 7).

Cancer cells acquire metastatic potential as a result of random mutations, genetic drift, and nonrandom selection (8), which can be manifested by genomic profile alteration. BRAF*^V600E^* and *TERT* promoter mutations were involved in tumorigenesis and metastasis of PTC. For instance, Xing et al. reported that PTC-specific mortality occurred in 22.7% (15/66) *vs.* 0.6% (4/629) in patients with genetic duet and neither respectively, suggesting independent prognostic value of these two types of somatic mutations (9). For BM alone, an 18-years retrospective study also revealed that majority of their 79 BM patient carry *TERT* promoter mutation, but failed to exhibit independent prognostic value due to the limited sample size (10). Moreover, a systematical genomic study was conducted in >10,000 patients with metastatic cancer recently, containing 93 metastatic PTC cases (11). Frequent mutations in BRAF*^V600E^* (66.7%) and *TERT* promoter (63.4%) were also observed (11). As a comparison, significant difference of mutation frequency in *TERT* promoter (~1%) rather than BRAF*^V600E^* (62%) was detected in patients with non-metastatic PTC in TCGA database (12), highlighting the prognostic value of *TERT* promoter mutations for PTC-related metastasis. Recently, trio samples (normal, primary tumor, and distant metastasis samples) from 14 patients were sequenced to identify shared and metastatic-specific genomic mutations (13). In another hand, PTC-related metastasis may be induced by primary high risk histological subtype of PTC (e.g., tall cell, columnar cell, and hobnail variants) or dedifferentiation, which can be determined by ^131^I uptake (14). For different types of thyroid cancer with varied differential states, comparison of their genomic profiles was determined through high-throughput sequencing. Higher mutation burden as well as frequent *TP53* mutations was identified in dedifferentiated thyroid cancer compared to PTC in public database including TCGA (12). The impact of *TP53* alterations on dedifferentiation were thus validated through transgenic mouse model (15). Additionally, increased hypomethylation of global Alu was in parallel with the dedifferentiation and progression of thyroid cancer in order from differentiated thyroid carcinoma (DTC), poorly differentiated thyroid carcinoma (PDTC), and anaplastic thyroid carcinoma (ATC) (16). Some interesting cases were also reported to illustrate the genomic profile of thyroid cancer dedifferentiation. For instance, whole genome sequencing was conducted for multiple samples from a single patient with synchronous follicular thyroid carcinoma (FTC), PDTC and ATC as well as regional lymph node metastasis, demonstrating the importance role of defects in DNA repair on clonal evolution of thyroid cancer (17). Although these cross-sectional studies provided the molecular basis, and partially supported the theory of stepwise dedifferentiation and metastasis process of thyroid cancer, a direct molecular evidence is still lacking with longitudinal genomic study.

In this study, we screened PTC patients with multi-steps of metastatic history in our hospital, and conducted whole exome sequencing (WES) on the available resected samples, aiming to illustrate the longitudinal genomic profile, address the metastatic seeding pattern, and provide the potential driver mutations in metastasis/dedifferentiation process of PTC.

## Methods

### Clinical Samples

Totally 12,458 PTC patients were treated in West China Hospital from 2000 to 2018. Screening was conducted with the following inclusion criteria: i. Conventional PTC as initial diagnosis; ii. Experience brain metastasis; iii. Lymph node metastasis prior to BM; and Exclusion criteria: i. Medullary/follicular/anaplastic thyroid cancer or mixed with these types; ii. No lymph node and brain metastasis sample available). Finally, stepwise resected samples were only available from one PTC case with brain metastasis, including specimen from four lymph nodes (lyn 1/2/3/4) and brain metastatic lesion (**Supplementary Figure 1**). Each sample was stained with Hematoxylin-eosin (**Supplementary Figure 2**).

### Genomic DNA Extraction, Whole-Exome Sequencing, and Genomic Analysis

After macrodissection, 3–5 slides of 10 μm sections from the FFPE blocks were resuspended in 200 μl deparaffinization solution (Qiagen, #1064343) and incubated at 56°C for 3 min. Genomic DNA were extracted following the GeneRead DNA FFPE Kit (Qiagen, #180134) manufacturer’s instruction and quantified through Qubit 2.0 fluorometer (Invitrogen). The extracted DNA was quantified through NanoDrop 2000 and agarose gel electrophoresis.

We conducted bioinformatics analyses followed our previous pipeline (18, 19). Briefly, sequencing libraries were generated using Agilent SureSelect Human All Exon kit (Agilent Technologies, CA, USA) kit and subject to Illumina Novaseq6000 for whole exome sequencing. The quality of raw reads was evaluated by FastQC (version 0.11.8). Subsequently, the qualified reads were mapped to human reference genome (GRCh37/hg19) using BWT algorithm as described previously (20). GATK4.0 best practice was applied to the aligned reads for duplication removal and base quality recalibration (21). Somatic mutations were called with Mutect2 and Strelka2 softwares (22). On the other hand, we have also computed the somatic copy number alterations *via* cnvkit (version 0.9.2). Tumor purities were estimated by ABSOLUTE software (23). The mutation signature was analyzed using DeconstructSigs to map the mutation to the COSMIC signatures (24). To reconstruct clone trees *via* the sequencing data, we employed treeomics to analyze the phylogenetic process of BM. Tumor purities and somatic mutations were used for the calculation. The genetic distance and Jaccard similarity coefficient between all pairs of samples in each patient were calculated using Treeomics as previous described (25).

### Establishment of Genetically Modified Cell Line

Humans PTC cell line (i.e., TPC-1) was gifted from the Hu Lab. Cell, which was cultured in RPMI 1640 (Hyclone) medium containing 10% FBS (PAN Seratech) and 1% Penicillin/Streptomycin (Gibco), grew at 37°C in 5% CO2 and 95% humidified air.

The sgRNA/Cas9 expression vector lentiCRISPRV2 was obtained from Addgene (Cambridge). The sequences for sgRNA targeting *TP53* gene was designed from (https://zlab.bio/guide-design-resources). TPC-1 was transfected with lipo3000 (Invitrogen), and treated with puromycin for 3 days. Allelic *TP53* knockout monoclones were selected and submitted to validation by Sanger sequencing (**Supplementary Figure 5A**).

Coding region of *CDK4* was inserted into overexpression vector (i.e., pCDH-CMV-MCS-EF1-puro) by homologous recombination, which was subsequently introduced point mutation (i.e., CDK4^R24C^) by using Q5 Site-Directed Mutagenesis Kit (NEB #E0552S). Lentivirus was used to infect TPC-1 and puromycin was used to screen and construct stable transfected cell lines with CDK4/CDK4^R24C^ overexpressing (**Supplementary Figures 5B, C**).

### Transwell Analysis

Cell migration experiments were performed using 24-well cell culture chambers (BD Biosciences) containing a PET membrane with 8 μm pores according to the manufactures instructions. TPC-1 was seeded at a density of 2 × 10^4^ cells/well in the upper chamber with culture medium (200 μl) alone, while the bottom of the plate was filled with culture medium (800 μl) supplemented with 10% FBS as a chemoattractant. After 24 h, cells that invaded the underside of the membrane were fixed with methanol and stained by crystal violet. The experiments were repeated for three times.

### Iodine Uptake Assay

Some 1 × 10^5^ cells/well were seeded in 6-well plates and incubated with 2 μCi carrier-free ^131^I or equal volume of HBSS for 24 h. After incubation, cells were washed twice with ice-cold HBSS and scraped from each well, and radioactivity was measured in a γ-counter. The radioactivity was normalized to the cell number for each cell type. The experiments were repeated for three times.

## Results

### Clinical Characteristics of the BM Patient and WES

**Figure 1:**
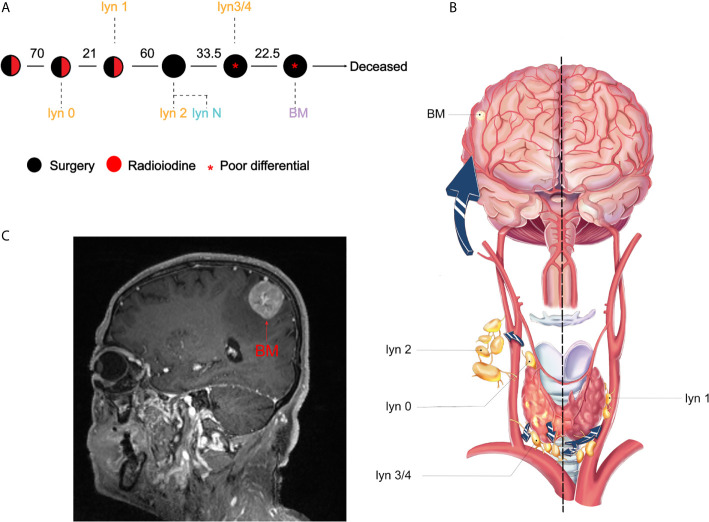
Illustration of treatment course and metastatic pattern PTC patient. **(A)** Clinical course is shown from the time of surgical resection of primary tumors to death. Circles represented treatment, black and red represented surgery and radioiodine, respectively. Red star indicated that poor differential component was found in the resected samples. The interval between two circles was shown in months; **(B)** Illustration of thyroid cancer and metastatic routes. lyn 0–4 represented the metastatic lymph nodes and BM represented brain metastasis; **(C)** Magnetic resonance imaging of the BM.

To comprehensively analyze the lineage relationship between lymph node (lyn) metastasis and BM, we retrospectively reviewed the clinical records of all thyroid cancer patients (n = 12,458) between 2000 and 2018. After stringent screening, 34 out of 7,135 (0.5%) cPTC patients experience brain metastasis, with no surgical indication for the majority of these patients. Only one case with stepwise resected samples was included in our study with available clinical samples (**Supplementary Figure 1**). The male patient was diagnosed at his 50 years-old as conventional papillary thyroid cancer (cPTC), which is the most differentiated subtype of thyroid cancer. The primary tumor was multifocal with partial fusion in right lobe, which invaded right strap muscle and underwent thyroidectomy in 2000. Thereafter four times of lymphadenectomy were performed because of locoregional recurrence. Five metastatic lymph nodes were resected, namely as lyn 0 to lyn 4 in chronological order, while a non-tumor Lymph node (lyn N) was obtained as control for genomic analysis (**Figure 1A**). lyn 0/2/3/4 located in the ipsilateral side with primary tumor, while lyn 1 located in the contralateral side (**Figure 1B**). Finally, the patient experienced intracranial metastasis (**Figure 1C**) and excision of craniocerebral tumor in 2018, with a total course of 18 years (**Figure 1A**). Notably, dedifferentiated thyroid cancer component was firstly found in recurrent metastatic lymph node in 2016 (i.e., lyn 3/4), while BM was diagnosed as metastatic poor differential thyroid cancer (**Supplementary Figure 2**).

Whole exome sequencing was performed in a total of six formalin-fixed paraffin-embedded (FFPE) samples acquired from metastatic lyn 1/2/3/4, BM and tumor-free lymph node (as reference), but not primary tumor and lyn 0 due to unavailability or low DNA quality. Tumor purity was also estimated by using ABSOLUTE, ranging from 30 to 99%, which was used to adjust for subsequent variant allele frequencies (VAF).

### Genomic Characteristics

**Figure 2:**
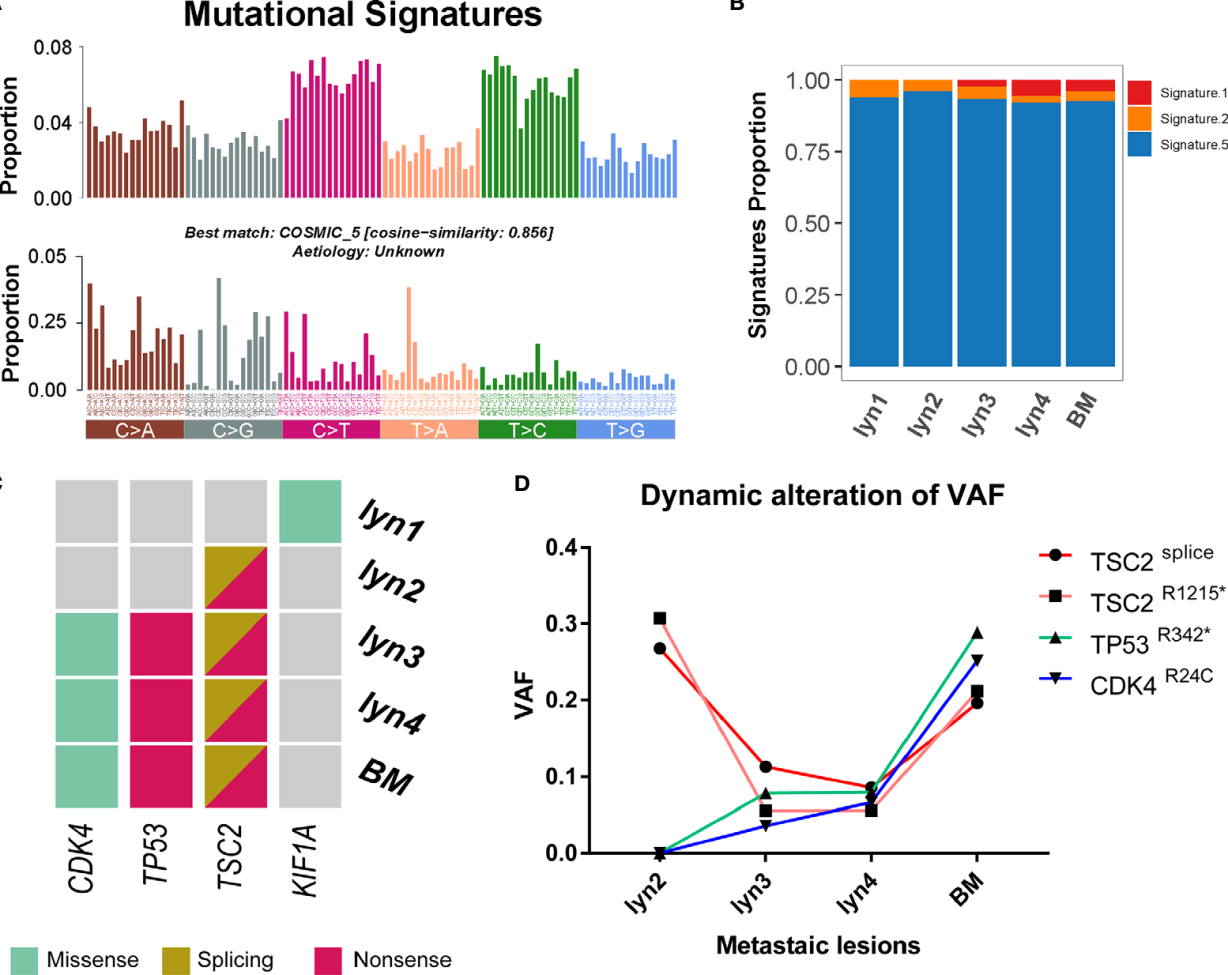
Landscape of mutation signature and potential driver mutations. **(A)** The 96-mutatinal classes of all somatic point mutations and the best matched signature; **(B)** Estimated signature component and relative ratio in each sample; **(C)** Heatmap of potential driver mutations in each sample; **(D)** Dynamic changes of variant allele frequencies of mutations in *TSC2*, *TP53* and *CDK4* along with the time course.

Taking lyn N as reference, somatic alterations were estimated by mature bioinformatics approaches. A total of 538 somatic mutations were identified (median of 2.2/Mb, ranging from 1.3 to 3.3/Mb), including 124 coding region or splicing sites, with a median of 39 (range from 32 to 54) in lymph node and brain metastasis (**Supplementary Table 1**). Spectrum of nucleotide case changes revealed an overrepresentation of C > T transitions for all samples (**Figure 2A**). Mutational signatures were estimated when considering context of the mutations, matching to the four signatures for thyroid cancer (26). Signature 5 were dominantly presented in all samples, which is consistent with previous report. On the other hand, Signature 1 were strictly presented in lyn 3/4 and BM (**Figures 2A, B**), suggesting their close relationship. Additionally, the number of Signature 1 mutations was annotated to correlates with age of cancer diagnosis, thus the proportion of Signature 1 consistently increased along with the age of our patient when he experienced lymph node metastasis to BM.

Mutation sites were shared among lyn 2/3/4 and BM in 16 genes (e.g., *TSC2*), and additional 9 among lyn3/4 and BM (e.g., *CDK4* and *TP53*), while no shared mutation was found between lyn 1 and other samples (**Supplementary Table 1**). Besides, 10 somatic mutations occurred in putative cancer driver genes (27), including one gene (i.e., *TSC2*) shared by lyn2/3/4 and BM, two shared by lyn 3/4 and BM (i.e., *TP53* and *CDK4*) (**Figure 2C**). In addition, impact of mutations on protein products were determined their damage effect, shared mutations of TSC2*^splicing^*, TSC2^R1215*^, TP53^R342*^ and CDK4^R24C^ were annotated as damaging, further establishing their functional potential in cancer development. Of note, two mutations in *TSC2* were shared by all metastatic samples except lyn 1, indicating the potential causal role of biallelic deficiency of *TSC2* in tumorigenesis. In another hand, although BRAF^V600E^ and *TERT* promoter hotspot mutations (i.e., C228T and C250T) were mostly reported as the key factor for PTC development, neither of these two common mutated genes was identified for either sample of this patient through both WES and Sanger sequencing approaches (**Supplementary Figure 3**), indicating tumorigenesis and metastasis of this case was not driven by these two common mutations. Considering the different dedifferentiated states of lyn 2 with lyn 3/4 and BM, and purity-adjusted variant allele frequencies of driver mutations among samples (**Figure 2D**), *TSC2* mutation was more likely to impact on tumorigenesis, while gradually enriched *TP53* and *CDK4* mutations may play an important role on dedifferentiation and subsequent metastasis.

Thereafter, WES-based somatic copy number alterations (SCNA) were also estimated, agreeing on the general diploid across all tumor samples with only a few focal SCNAs except haploid for chromosome X (**Supplementary Figure 4A**). lyn 1 have distinct broad gain/loss of chromosomes compared to other metastasis samples, (e.g., loss of entire chromosome 9 and Y), confirming its different origination of primary tumor from other metastatic samples. In another hand, no specific cancer-related gene was affected by foci SCNA shared by lyn 3/4 and BM (**Supplementary Figure 4B**).

### Longitudinal Metastatic Pattern of Brain Metastasis

**Figure 3:**
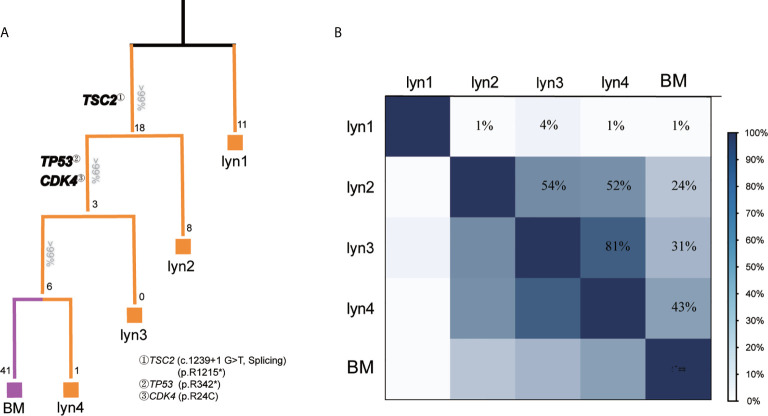
longitudinal evolution pattern and similarity among metastatic lesions. **(A)** Phylogenetic trees were reconstructed with phylogenomic methods and scaled to demonstrate the metastatic pattern. Numbers on the top of each branch represented the number of shared somatic coding/spicing mutations, numbers at the end of each branch represented acquired private mutations, potential driver gene mutations were illustrated in bold; **(B)** Genetic similarity analysis between each metastatic lesion.

**Figure 4:**
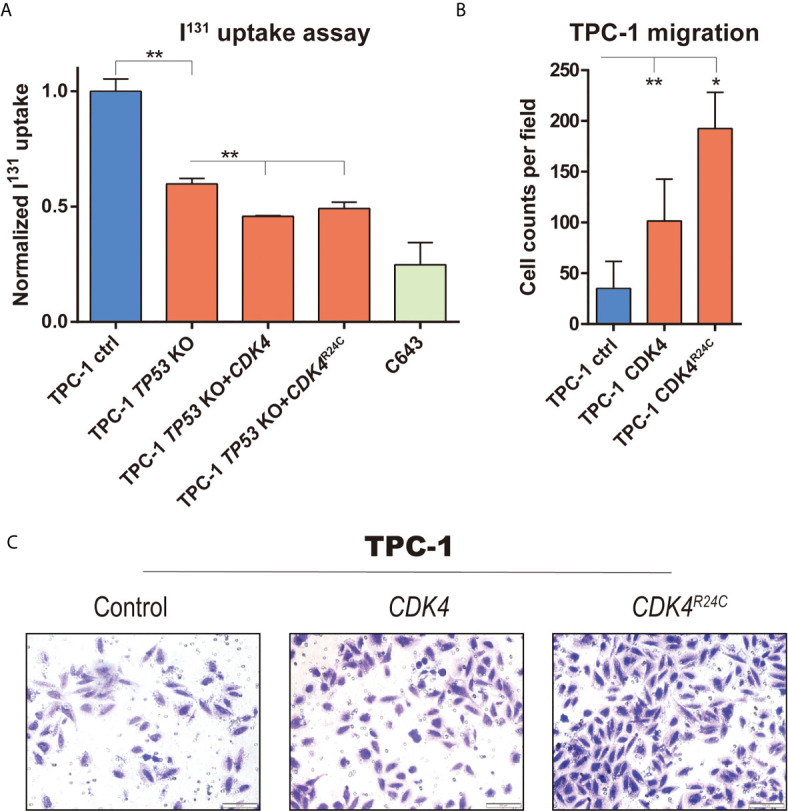
The impact of mutations in *TP53* and *CDK4* on migration and differentiation of PTC cells. **(A)** ^131^I uptake in TPC-1 cell with different modification (*TP53* knockout, and *CDK4*/*CDK4* ^R24C^ overexpression), with C643 as positive control; **(B)** Representative image capture and **(C)** quantification of transwell assay in TPC-1 and CDK4/CDK4^R24C^ overexpression. *, P value < 0.05; **, P value < 0.01.

To further analyze the metastatic pattern of this patient, we reconstructed phylogenetic tree to infer the metastatic seeding pattern (**Figure 3A**), which well fitted the time course of metastasis. Detailedly, lyn 1 metastasis originated from a distinct primary tumor, and carried missense mutations in a putative driver gene (i.e., *KIF3A*) (**Figure 2C**). TSC2^splicing^ and TSC2^R1215*^ located in the trunk of phylogenetic tree for all samples except lyn 1 (**Figure 3A**), suggesting its potential role on tumorigenesis of another primary PTC of this patient. Dedifferentiation Subclone raised from the trunk, but pathologic evidence of dedifferentiation was observed in lyn 3/4 and BM but not lyn 2 (**Supplementary Figure 2**). Not surprisingly, lyn 3/4 and BM, which shared TP53^R342*^ and CDK4^R24C^ mutations, located in a separated branch apart from lyn 2, suggesting these two functional mutations may contribute to the dedifferentiation process of metastasis, particularly facilitate the tumor cells seeding into the brain. Inference of similarities among tumor samples revealed high level of heterogeneity between lyn 1 and other metastatic lesions (lyn 2/3/4 and BM) (**Figure 3B**), indicating they may originate from different subclones or cancer foci of the primary tumor, which was consistent previous report (20). In another hand, the highest similarity was observed between lyn 4 and BM (43%) (**Figure 4B**), supporting the fact of close genetic distance between these two metastatic lesions, and derivation of BM from lyn 4.

### Impact of *TP53* and *CDK4* Mutation on Metastasis of PTC

According to the reports, one third of thyroid cancer metastases lost ability to concentrate iodine through dedifferentiation, and thus resistant to radioiodine therapy (28, 29), while Impact of *TP53* alterations on PTC dedifferentiation has been well established and evaluated in mouse model with thyroid cancer (15). Considering the appearance of TP53 ^R342*^ and CDK4^R24C^ in the late stage of metastasis, we speculated that functionally TP53^R342*^ mutation may probably contribute to the dedifferentiation process and subsequent radioiodine resistance. Therefore, we constructed a CRISPR/CAS9 based *TP53* knockout in TPC-1 (a PTC cell line with neither *BRAF* ^V600E^ nor *TERT* mutation) to represent *TP53* nonsense mutation, and evaluated its impact on ^131^I uptake. As positive control, the ATC cell line (i.e., C643) has a significant lower uptake of ^131^I compared to that in TPC-1 cells. Interestingly, knocking-out of *TP53* also significantly reduced the uptake of ^131^I in TPC-1, which is consistent with our hypothesis (**Figure 4A**). Moreover, introduction of CDK4 and CDK4^R24C^ in TP53 knockout cells can further reduce uptake of ^131^I, indicating the possible synergistic effect of *TP53* deficiency and *CDK4* mutation in dedifferentiation of PTC (**Figure 4A**).

In another hand, although metastatic ability of melanoma can be induced by CDK4^R24C^ mutation through migration assay (30), such impact has never been estimated in PTC. Therefore, overexpression of CDK4 and CDK4^R24C^ was introduced into TPC-1 cells. Compared to the empty control, introduction of either CDK4 or CDK4^R24C^ significantly increased the migration ability of TPC-1 cells (**Figure 4B**). Moreover, overexpression of CDK4^R24C^ contributes to a significantly higher increased fold of migrated cells than that of CDK4 (**Figure 4C**), suggesting the enhanced impact of CDK4^R24C^ on metastasis of PTC, and the possible contribution of such mutation to stepwise metastasis of our cPTC patient.

## Discussion

PTC exhibits high differentiation and low risk of metastasis, and specific genomic alterations were considered to be involved in its dedifferentiation and metastasis process. With cross-sectional studies, multiple genes were identified to be restrictedly mutated in the metastatic lesions (e.g., *SIN3A*, *RBBP1* and *CHD4*) (13), but can’t provide the direct molecular evidence for stepwise dedifferentiation and metastasis process. In another hand, brain metastasis is very rare, especially for cPTC, and even harder to obtain the samples from different steps of metastatic process. Up to now, only two PTC cases with BM has been sequenced in previous cross-sectional studies, with one have metastatic samples only (11), and one with trio samples (normal control, primary and BM) (13). In this study, we fortunately got surgical specimen and biopsies from one cPTC case with BM and stepwise resected metastatic lymph nodes. Therefore, our longitudinal genomic analyses not only demonstrating the phylogenetic evolution of PTC with BM for the first time, but also providing molecular basis of dedifferentiation and metastasis for cPTC.

Overall, the divergence and similarity among metastatic sites well fitted the time cross and seeding pattern of the metastasis. Since similar mutational pattern of driver genes may speculate the same origin (31), heterogeneity of the lyn 1 and other metastatic lesions (lyn 2/3/4 and BM), illustrated as none overlapping mutations and 0% of similarity, implies that this patient probably carry at least two primary PTC foci. In addition, genomic distinct metastatic lymph nodes were also separated apart from each other in terms of physical locations. According to the clinical record of this patient, the first metastatic lymph node (i.e., lyn 0) located at the same side with lyn 2/3/4, suggesting the possibility of lyn 0 as the metastatic origin of lyn2/3/4 and BM. However, the direct genomic evidence is lacking because the genetic profile of primary tumor and the lyn 0 are not available due to long storage (over 12–18 years) induced poor DNA quality. Nevertheless, it is considered that functional driver gene mutations identified by a single biopsy may be shared by all primary and metastases within a patient (31). Therefore, the genomic profile of all metastatic lesions may infer that of primary tumor. Moreover, metastatic samples with shared trunk (e.g., TSC2*^splicing^* and TSC2^R1215*^) and branch mutations (e.g., TP53^R342*^ and CDK4^R24C^) may also illustrate the genomic basis of stepwise metastasis and dedifferentiation.

Two mutations of *TSC2* were identified in all differentiated and dedifferentiated samples except lyn 1, suggesting its role on tumorigenesis of PTC. Actually, *TSC2* encodes a tumor suppressor, which is capable of stimulating specific GTPases (32, 33) and negatively regulate mTOR signaling (34). Somatic *TSC2* mutation contributes to tumorigenesis, including renal cancer (35). Therefore, we speculated that these two mutations may inactivate both alleles of *TCS2*, which unfortunately cannot be experimentally validated due to the long distance of these two mutations. Moreover, a recent genomic landscape of 10,000 metastatic tumor revealed that *TSC2* missense mutations were presented in 294 patients with different types of cancer, but only one mutation was identified in 93 patients with PTC-related metastasis (11). Interestingly, this patient is coincidentally the sole case who experienced brain metastasis, suggesting the *TSC2* mutations may increase the risk of brain metastasis of PTC.

In another hand, p53 (encoded by *TP53*) and CDK4 are well-established tumor suppressor and oncogene, respectively, involving in cell cycle checkpoint of G1/S phase. Activity of CDK4 can be inhibited by p21, which is the direct downstream target of p53 (36). Gain of function mutation in *CDK4* is commonly observed in both germline and somatic level of cancers, with the most frequent pattern of CDK4^R24C^ (37–41). Such mutation can renders CDK4 insensitive to INK4 inhibitors (e.g., p16^INK4A^) through preventing their binding (42), and induce development of multiple types of tumor in knock-in mouse model (43). Importantly, significant increased migration ability is gained after introducing CDK4^R24C^ into melanoma cells (30), which is consistent with our experiment result in PTC cells, suggesting this mutation may play contribute to metastasis of our patient with BM. However, no *CDK4* mutation has been identified in any types of thyroid cancer so far according to the public resource (12, 44, 45), consistent with the rarity of cPTC-related brain metastasis. Meanwhile, loss of function nonsense mutation in *TP53* (i.e., TP53^R342*^) arise together with CDK4^R24C^, with the same trend of increased content from lyn 3 to BM (**Figure 2D**), indicating they are mutated in the same subclone. The important roles of *TP53* on tumor have been well-established, and a series of studied (e.g., TCGA project) also indicated the rare *TP53* mutation rate in PTC (<1%) compared to ATC (>30%) (45, 46), and contribution of *TP53* loss to the dedifferentiation of differential thyroid cancer (15, 47, 48), which is also experimentally validated in our study. Indeed, mutation frequency of different types of thyroid cancer varied in parallel with their differentiated states, with the highest *TP53* mutation rate observed in ATC (the poorest differentiated subtype of thyroid cancer), while lowest in cPTC (the most differentiated subtype) (12, 44), further supporting the contribution of *TP53* deficiency to dedifferentiation of thyroid cancer. Therefore, based on current public research findings as well as our experimental result, it is considered that combination of CDK4^R24C^ and TP53^R342*^ mutations may contribute to the metastasis and dedifferentiation process of our cPTC case.

Several limitations should be noticed in this study. First, brain metastasis is extremely rare in cPTC (0.5% in our patient cohort), mostly with no surgical indication, thus it is very difficult to obtain the clinical samples of brain metastasis. In this case, samples from only one patient were sequenced in this study, and involvement of *TP53* and *CDK4* mutations in the dedifferentiation process of cPTC should be validated in a large cohort. Second, due to the long duration of brain metastasis free survival of this patient (20 years from the primary surgery to brain metastasis), tumor samples were not available from the primary cPTC lesion, but several lymph nodes metastasis. The lack of primary tumor sample prevented us to determine the trunk mutations of this patient, whether bi-allelic *TSC2* mutations were involved in tumorigenesis or dedifferentiation should be determined in other cohorts or functional assay. Third, because we can only conduct WES with FFPE samples, it is impossible for us to detect the fusion genes commonly reported in thyroid cancer (e.g., CCDC6-RET and NTRK fusions) (49), which could be the driver genomic event in the primary tumor of our patient and shared by all the metastatic samples.

In conclusion, we performed a longitudinal genomic analysis for a rare case of cPTC with brain metastasis, and revealed its molecular metastatic seeding pattern. Functionally mutations in *TSC2*, *CDK4* and *TP53* may contribute to the risk of development, dedifferentiation and metastasis of PTC.

## Data Availability Statement

The original contributions presented in the study are publicly available. This data can be found here: (https://bigd.big.ac.cn/gsa-human/browse/HRA000283).

## Ethics Statement

The studies involving human participants were reviewed and approved by the Institutional Review Board of West China Hospital of Sichuan University (No. 2020-888). The ethics committee waived the requirement of written informed consent for participation.

## Author Contributions

HX, JZ, and YS designed and supervised this study. XL, QH, ZX, YL, YW, and LZ conducted the experiments and data analyses. HL and HX interpreted the data. HL, YQ, YJ, LS, HC, TW and ZL collected the clinical samples and information. LD, LY, and WZ vouch for the data and the analysis. HL and HX contributed to the conception of the study and drafted the manuscript. All authors contributed to the article and approved the submitted version.

## Funding

This work was supported by (1) National Natural Science Foundation of China (No. 81903735); (2) China Postdoctoral Science Foundation (2019M653416); (3) Sichuan Science and Technology Program (2019YFS0333); (4) 1.3.5 Project for Disciplines of Excellence, West China Hospital, Sichuan University (No. ZYJC18035, No. ZYYC20003, No. ZYJC18025, No. ZYJC21006, and ZYJC21024); (5) International Cooperation Project of Chengdu Municipal Science and Technology Bureau (No. 2020-GH02-00017-HZ); (6) Post-Doctor Research Project, West China Hospital, Sichuan University (2018HXBH004).

## Conflict of Interest

The authors declare that the research was conducted in the absence of any commercial or financial relationships that could be construed as a potential conflict of interest.

## Supplementary Material

Supplementary Figure 1 Flowchart of patients’ inclusion/exclusion.

Supplementary Figure 2 Hematoxylin-eosin staining of each metastatic sample for determination of differentiation status.

Supplementary Figure 3 Sanger sequencing detection of *BRAF*, and *TERT* promoter. The black arrow indicated the hot spot of mutated *BRAF* and *TERT* promoter in previous reports.

Supplementary Figure 4 Illustration of WES-based SCNA. **(A)** SCNA landscape of each sample; **(B)** estimated foci SCNA.

Supplementary Figure 5 Construction of *TP53* knockout and CDK4/CDK4^R24C^ overexpression. **(A)** gRNA sequencing information and validation of TPC-1 monoclone with *TP53* knockout; **(B)** construction of CDK4/CDK4^R24C^ overexpression plasmid; (C) evaluation of CDK4/CDK4^R24C^ overexpression in TPC-1 cells through quantitative-PCR.
