# Supplementary figures and images for: Longitudinal Genomic Evolution of Conventional Papillary Thyroid Cancer With Brain Metastasis
Source: Front Oncol. 2021 Jun 23;11:620924. doi: 10.3389/fonc.2021.620924 (PMC8260944; doi:10.3389/fonc.2021.620924)

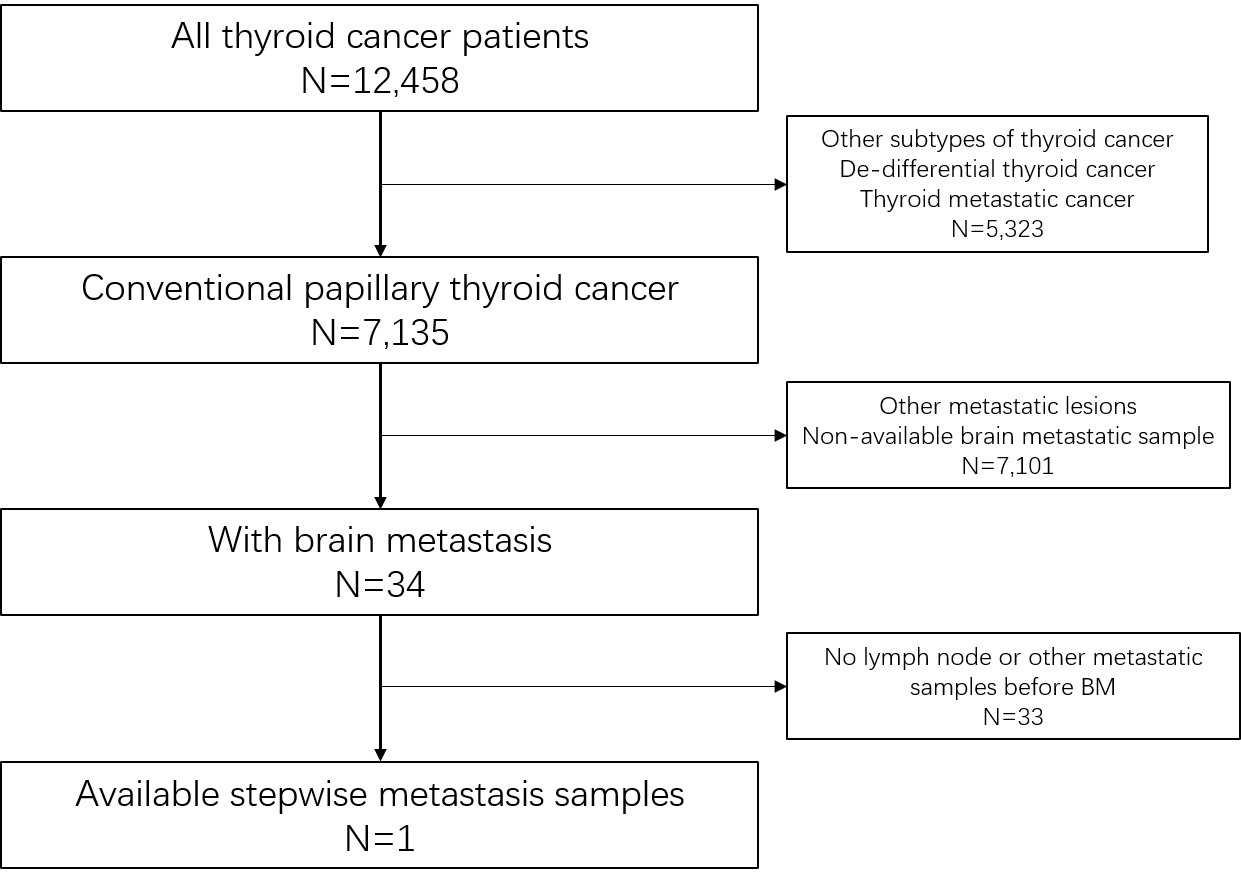

Supplement: Supplementary Figure 1 — Flowchart of patients’ inclusion/exclusion. [file Image_1.tif]

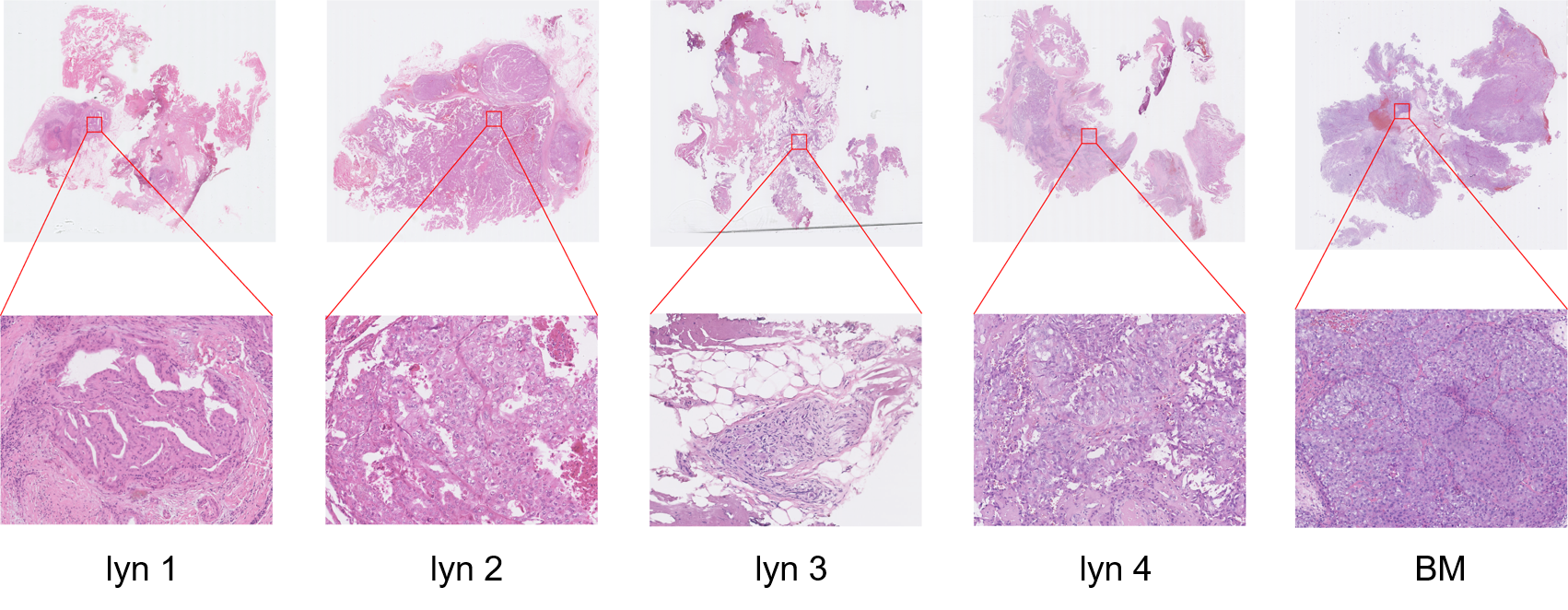

Supplement: Supplementary Figure 2 — Hematoxylin-eosin staining of each metastatic sample for determination of differentiation status. [file Image_2.tif]

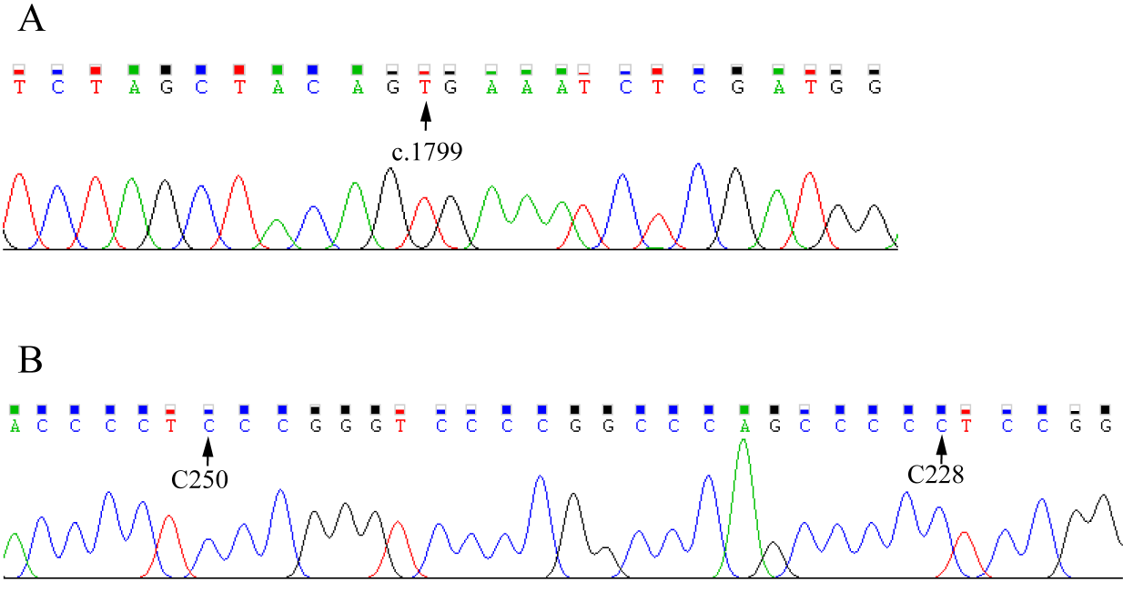

Supplement: Supplementary Figure 3 — Sanger sequencing detection of BRAF, and TERT promoter. The black arrow indicated the hot spot of mutated BRAF and TERT promoter in previous reports. [file Image_3.tif]

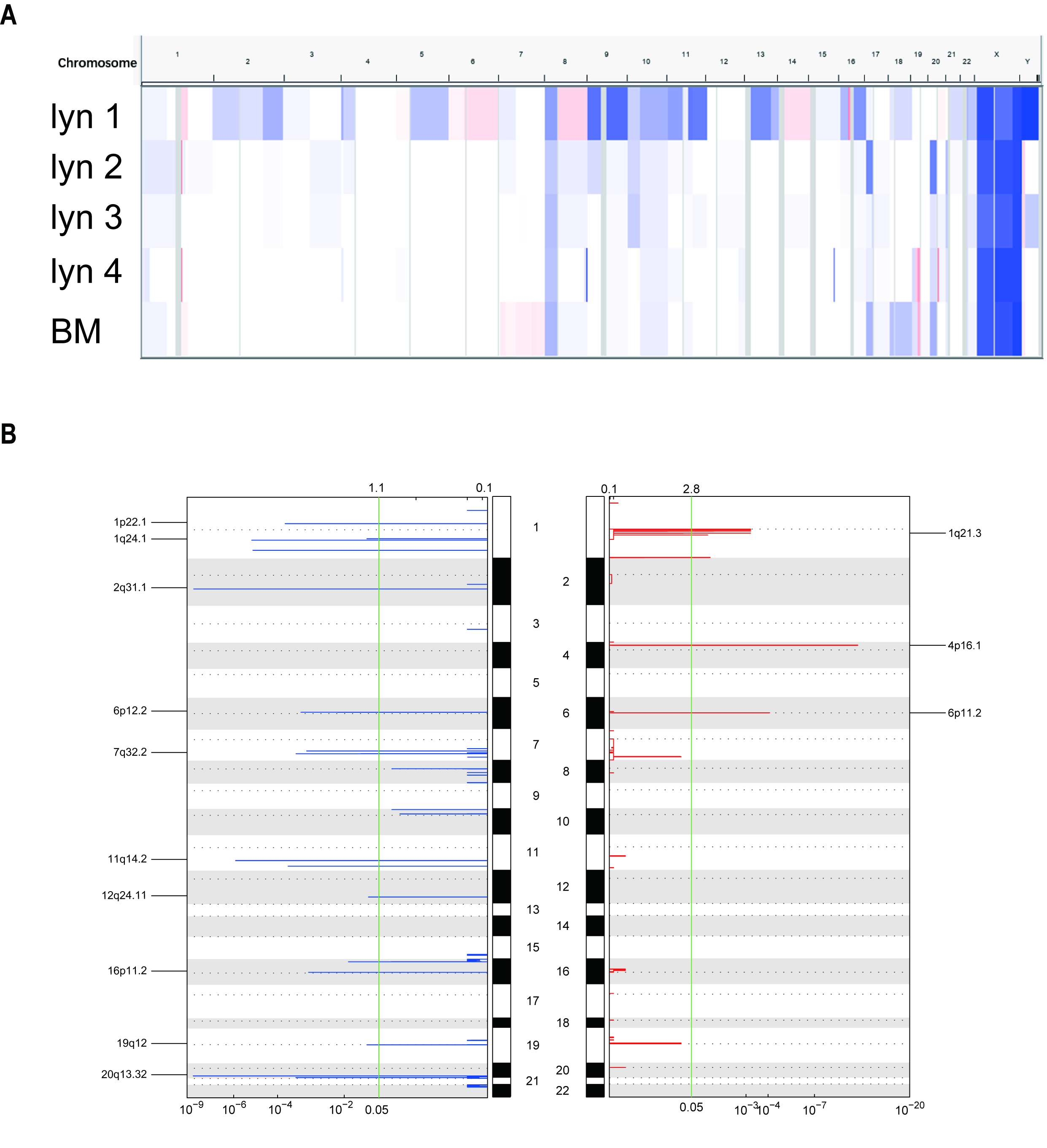

Supplement: Supplementary Figure 4 — Illustration of WES-based SCNA. (A) SCNA landscape of each sample; (B) estimated foci SCNA. [file Image_4.tif]

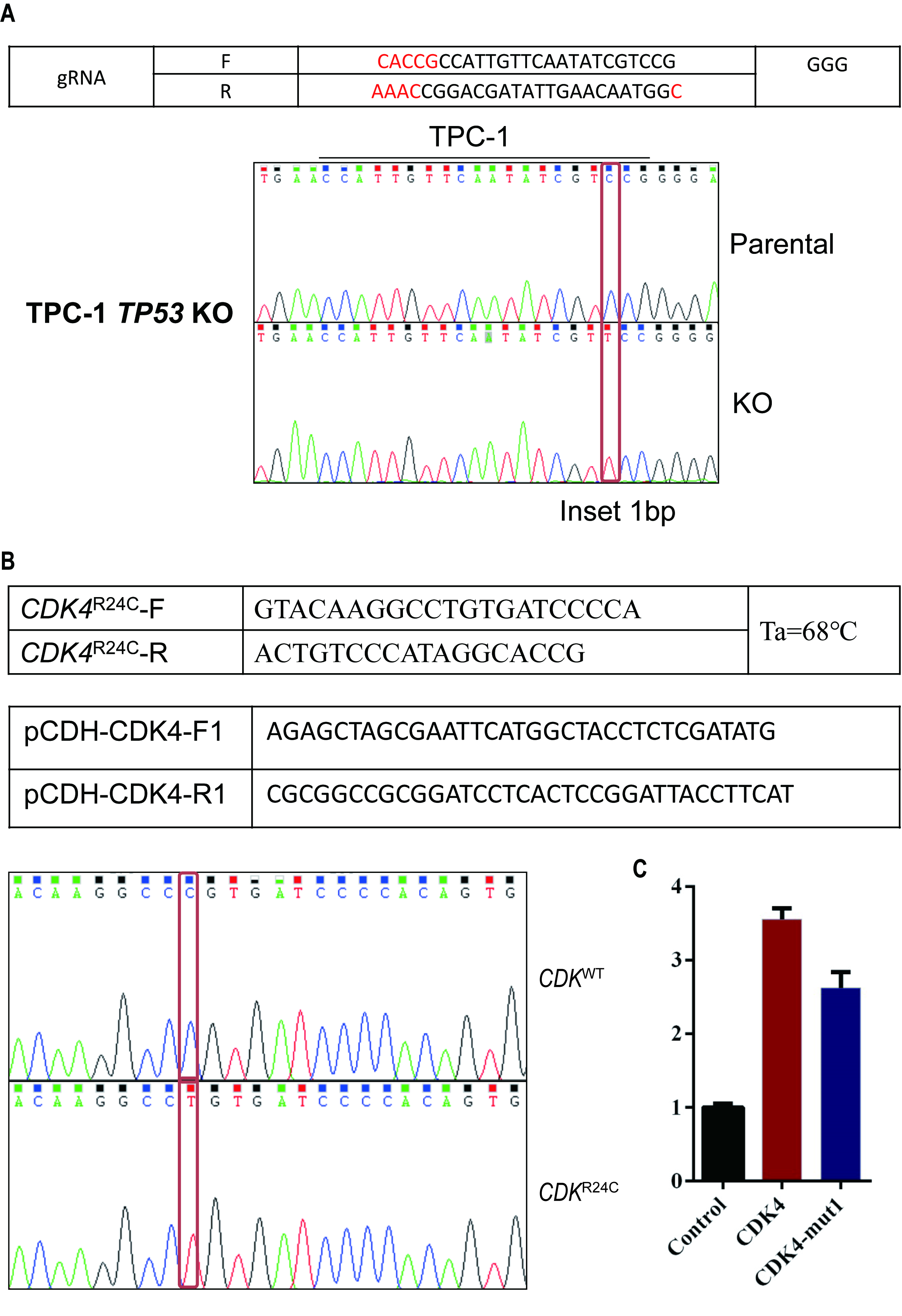

Supplement: Supplementary Figure 5 — Construction of TP53 knockout and CDK4/CDK4R24C overexpression. (A) gRNA sequencing information and validation of TPC-1 monoclone with TP53 knockout; (B) construction of CDK4/CDK4R24C overexpression plasmid; (C) evaluation of CDK4/CDK4R24C overexpression in TPC-1 cells through quantitative-PCR. [file Image_5.tif]
