# Supplementary material for: Longitudinal Genomic Evolution of Conventional Papillary Thyroid Cancer With Brain Metastasis
Source: Front Oncol. 2021 Jun 23;11:620924. doi: 10.3389/fonc.2021.620924 (PMC8260944; doi:10.3389/fonc.2021.620924)
Supplement: Supplementary file 6 [file Table_1.docx]

Supplementary Table 1: Summary of all mutations in coding region and splicing site

| **Chr** | **StartPosition** | **EndPosition** | **RefAllele** | **AltAllele** | **GeneSymbol** | **mution type** | **AA change** | **Driver** | **Phylogeny** | **shared samples** |
| --- | --- | --- | --- | --- | --- | --- | --- | --- | --- | --- |
| 16 | 2120580 | 2120580 | G | T | TSC2 | splicing |  | TRUE | Shared | lyn2 lyn3 lyn4 BM |
| 16 | 2134598 | 2134598 | C | T | TSC2 | nonsense | p.R1344X | TRUE | Shared | lyn2 lyn3 lyn4 BM |
| 14 | 76071924 | 76071924 | A | G | FLVCR2 | missense | p.D4G | FALSE | Shared | lyn2 lyn3 lyn4 BM |
| 16 | 69681300 | 69681300 | C | A | NFAT5 | missense | p.S208Y | FALSE | Shared | lyn2 lyn3 lyn4 BM |
| 7 | 128520041 | 128520041 | G | A | KCP | Silent | p.F1159F | FALSE | Shared | lyn2 lyn3 lyn4 BM |
| 19 | 39866413 | 39866413 | C | T | SAMD4B | missense | p.T264M | FALSE | Shared | lyn2 lyn3 lyn4 BM |
| 7 | 34192732 | 34192732 | C | T | BMPER | Silent | p.Y635Y | FALSE | Shared | lyn2 lyn3 lyn4 BM |
| 19 | 13050040 | 13050043 | AAAG | A | CALR | inframe del | p.62_63del | FALSE | Shared | lyn2 lyn3 lyn4 BM |
| 19 | 13050044 | 13050044 | A | T | CALR | missense | p.D63V | FALSE | Shared | lyn2 lyn3 lyn4 BM |
| 14 | 81610265 | 81610265 | A | AATTGCCAAGAGG | TSHR | inframe Ins | p.K621KIAKR | FALSE | Shared | lyn2 lyn3 lyn4 BM |
| 10 | 28906621 | 28906621 | A | G | WAC | Silent | p.G549G | FALSE | Shared | lyn2 lyn3 lyn4 BM |
| 3 | 154032924 | 154032924 | G | GC | DHX36 | FrameShift ins | p.Q172fs | FALSE | Shared | lyn2 lyn3 lyn4 BM |
| 1 | 35569951 | 35569952 | TG | T | ZMYM1 | FrameShift del | p.C159fs | FALSE | Shared | lyn2 lyn3 lyn4 BM |
| 9 | 286628 | 286628 | G | A | DOCK8 | Silent | p.P108P | FALSE | Shared | lyn2 lyn3 lyn4 BM |
| 17 | 45890670 | 45890670 | C | T | OSBPL7 | missense | p.E567K | FALSE | Shared | lyn2 lyn3 lyn4 BM |
| 1 | 35569953 | 35569953 | C | A | ZMYM1 | nonsense | p.C159X | FALSE | Shared | lyn2 lyn3 lyn4 BM |
| 7 | 44185197 | 44185197 | C | T | GCK | Silent | p.A383A | FALSE | Shared | lyn2 lyn3 lyn4 BM |
| 17 | 7574003 | 7574003 | G | A | TP53 | nonsense | p.R303X | TRUE | Shared | lyn3 lyn4 BM |
| 14 | 23596513 | 23596513 | A | C | SLC7A8 | missense | p.V270G | FALSE | Shared | lyn3 lyn4 BM |
| 21 | 40649233 | 40649233 | T | A | BRWD1 | missense | p.M350L | FALSE | Shared | lyn3 lyn4 BM |
| 12 | 124421847 | 124421847 | A | G | CCDC92 | missense | p.S252P | FALSE | Shared | lyn3 lyn4 BM |
| X | 47086299 | 47086299 | C | T | CDK16 | missense | p.R382C | FALSE | Shared | lyn3 lyn4 BM |
| 7 | 138946109 | 138946109 | G | T | UBN2 | missense | p.K339N | FALSE | Shared | lyn3 lyn4 BM |
| 7 | 21789308 | 21789308 | A | G | DNAH11 | missense | p.N2896D | FALSE | Shared | lyn3 lyn4 BM |
| 13 | 51943151 | 51943152 | CA | C | INTS6 | FrameShift del | p.M622fs | FALSE | Shared | lyn3 lyn4 BM |
| 12 | 58145431 | 58145431 | G | A | CDK4 | missense | p.R24C | TRUE | Shared | lyn3 lyn4 BM |
| X | 55028844 | 55028844 | C | T | APEX2 | Silent | p.L134L | FALSE | Shared | lyn4 BM |
| 7 | 156480809 | 156480809 | G | C | LMBR1 | nonsense | p.Y282X | FALSE | Shared | lyn4 BM |
| 8 | 21829450 | 21829450 | C | T | XPO7 | nonsense | p.Q164X | FALSE | Shared | lyn4 BM |
| 12 | 2977808 | 2977808 | C | T | FOXM1 | missense | p.R256H | FALSE | Shared | lyn4 BM |
| 17 | 29855641 | 29855641 | A | G | RAB11FIP4 | missense | p.I500V | FALSE | Shared | lyn4 BM |
| 3 | 11886571 | 11886571 | C | G | TAMM41 | splicing |  | FALSE | Shared | lyn4 BM |
| 1 | 35569950 | 35569951 | TG | T | ZMYM1 | frameshift del | p.C84fs | FALSE | Shared | lyn4 BM |
| 6 | 47647997 | 47647997 | T | A | ADGRF2 | missense | p.I153K | FALSE | Private | lyn1 |
| X | 112054602 | 112054602 | C | T | AMOT | missense | p.R62H | FALSE | Private | lyn1 |
| 6 | 34985252 | 34985252 | C | T | ANKS1A | missense | p.H476Y | FALSE | Private | lyn1 |
| 6 | 42072861 | 42072861 | C | T | C6orf132 | missense | p.S930N | FALSE | Private | lyn1 |
| 8 | 145754243 | 145754243 | G | A | C8orf82 | missense | p.R20W | FALSE | Private | lyn1 |
| 11 | 64951020 | 64951020 | G | C | CAPN1 | missense | p.G138A | FALSE | Private | lyn1 |
| 12 | 120210599 | 120210599 | A | T | CIT | missense | p.I686N | FALSE | Private | lyn1 |
| 2 | 208989187 | 208989187 | A | C | CRYGD | splicing |  | FALSE | Private | lyn1 |
| 22 | 42539481 | 42539481 | G | A | CYP2D7 | Silent | p.R4R | FALSE | Private | lyn1 |
| 15 | 45401081 | 45401081 | T | C | DUOX2 | missense | p.D435G | FALSE | Private | lyn1 |
| 11 | 34502395 | 34502395 | C | G | ELF5 | missense | p.E104Q | FALSE | Private | lyn1 |
| X | 153577882 | 153577882 | A | G | FLNA | missense | p.V2535A | TRUE | Private | lyn1 |
| 13 | 29008324 | 29008324 | G | A | FLT1 | missense | p.R183C | FALSE | Private | lyn1 |
| 2 | 241658581 | 241658581 | G | C | KIF1A | missense | p.L1603V | TRUE | Private | lyn1 |
| 6 | 136732791 | 136732791 | G | A | MAP7 | missense | p.R71W | FALSE | Private | lyn1 |
| 1 | 211848796 | 211848796 | T | A | NEK2 | missense | p.E9V | FALSE | Private | lyn1 |
| 1 | 156640160 | 156640160 | C | T | NES | missense | p.G1274S | FALSE | Private | lyn1 |
| 14 | 51225007 | 51225007 | T | A | NIN | missense | p.Q914L | FALSE | Private | lyn1 |
| 2 | 73429916 | 73429916 | C | T | NOTO | missense | p.A41V | FALSE | Private | lyn1 |
| 6 | 17626089 | 17626089 | G | A | NUP153 | missense | p.T1315I | FALSE | Private | lyn1 |
| 11 | 5373606 | 5373606 | A | G | OR51B6 | missense | p.Y290C | FALSE | Private | lyn1 |
| 1 | 43220546 | 43220546 | G | A | P3H1 | missense | p.R447W | FALSE | Private | lyn1 |
| 19 | 1486989 | 1486989 | C | T | PCSK4 | missense | p.D311N | FALSE | Private | lyn1 |
| 11 | 63342457 | 63342457 | A | G | PLA2G16 | missense | p.I150T | FALSE | Private | lyn1 |
| 4 | 128807028 | 128807028 | A | G | PLK4 | missense | p.H127R | FALSE | Private | lyn1 |
| 3 | 135820866 | 135820866 | G | A | PPP2R3A | missense | p.R361H | FALSE | Private | lyn1 |
| 9 | 86616961 | 86616961 | C | T | RMI1 | missense | p.H354Y | FALSE | Private | lyn1 |
| 3 | 47098528 | 47098528 | T | C | SETD2 | missense | p.H2205R | TRUE | Private | lyn1 |
| 1 | 153750718 | 153750718 | C | T | SLC27A3 | missense | p.R543C | FALSE | Private | lyn1 |
| 1 | 85648184 | 85648184 | G | A | SYDE2 | missense | p.A714V | FALSE | Private | lyn1 |
| 10 | 75407224 | 75407224 | G | A | SYNPO2L | missense | p.P505L | FALSE | Private | lyn1 |
| 1 | 152083297 | 152083297 | C | T | TCHH | missense | p.R799K | FALSE | Private | lyn1 |
| 11 | 78600940 | 78600940 | C | T | TENM4 | missense | p.R325Q | FALSE | Private | lyn1 |
| 4 | 154556620 | 154556620 | C | G | TMEM131L | missense | p.A1484G | FALSE | Private | lyn1 |
| 12 | 88566458 | 88566458 | T | C | TMTC3 | missense | p.Y379H | FALSE | Private | lyn1 |
| 8 | 72973957 | 72973957 | C | T | TRPA1 | missense | p.G283R | FALSE | Private | lyn1 |
| 2 | 179437867 | 179437867 | C | T | TTN | missense | p.R24331H | FALSE | Private | lyn1 |
| 10 | 97917489 | 97917489 | A | T | ZNF518A | Silent | p.P470P | FALSE | Private | lyn1 |
| 19 | 38229901 | 38229901 | G | A | ZNF573 | missense | p.P495L | FALSE | Private | lyn1 |
| 17 | 80790203 | 80790203 | T | C | ZNF750 | missense | p.H43R | TRUE | Private | lyn1 |
| 1 | 151773761 | 151773761 | C | A | LINGO4 | missense | p.V474L | FALSE | Private | lyn1 |
| 3 | 113004360 | 113004361 | GC | G | BOC | frameshift del | p.A1037fs | FALSE | Private | lyn1 |
| 4 | 155158248 | 155158248 | A | G | DCHS2 | missense | p.V2064A | FALSE | Private | lyn1 |
| 5 | 131973889 | 131973889 | C | T | RAD50 | nonsense | p.R1198X | FALSE | Private | lyn1 |
| 5 | 140564382 | 140564382 | C | T | PCDHB16 | nonsense | p.Q750X | FALSE | Private | lyn1 |
| 7 | 15430483 | 15430486 | TAAG | T | AGMO | inframe del | p.241_241del | FALSE | Private | lyn1 |
| 10 | 76735886 | 76735887 | AT | A | KAT6B | missense | p.I598fs | TRUE | Private | lyn1 |
| 14 | 20666175 | 20666175 | C | CA | OR11G2 | missense | p.C227fs | FALSE | Private | lyn1 |
| 14 | 23744797 | 23744800 | ACAT | A | HOMEZ | inframe del | p.546_547del | FALSE | Private | lyn1 |
| 16 | 31383055 | 31383055 | C | T | ITGAX | nonsense | p.R704X | FALSE | Private | lyn1 |
| 16 | 67337072 | 67337073 | CG | C | KCTD19 | missense | p.E207fs | FALSE | Private | lyn1 |
| 17 | 3801372 | 3801375 | AAGC | A | P2RX1 | inframe del | p.354_355del | FALSE | Private | lyn1 |
| 17 | 36485655 | 36485655 | G | A | GPR179 | missense | p.P1266L | FALSE | Private | lyn1 |
| 19 | 52569606 | 52569607 | CT | C | ZNF841 | missense | p.E510fs | FALSE | Private | lyn1 |
| 2 | 32696129 | 32696129 | A | G | BIRC6 | missense | p.R2177G | FALSE | Private | lyn2 |
| 6 | 75887612 | 75887612 | G | A | COL12A1 | missense | p.T735I | FALSE | Private | lyn2 |
| X | 100078386 | 100078386 | A | G | CSTF2 | missense | p.E138G | FALSE | Private | lyn2 |
| 2 | 196722167 | 196722167 | A | G | DNAH7 | missense | p.I2783T | FALSE | Private | lyn2 |
| 6 | 159672346 | 159672346 | C | T | FNDC1 | missense | p.T1616M | FALSE | Private | lyn2 |
| 17 | 3628320 | 3628320 | A | G | HASPIN | missense | p.K364R | FALSE | Private | lyn2 |
| 19 | 7184370 | 7184370 | A | G | INSR | missense | p.C311R | FALSE | Private | lyn2 |
| 5 | 145547822 | 145547822 | C | T | LARS | missense | p.A47T | FALSE | Private | lyn2 |
| 4 | 41673569 | 41673569 | A | T | LIMCH1 | splicing |  | FALSE | Private | lyn2 |
| 2 | 15613345 | 15613345 | C | T | NBAS | splicing |  | FALSE | Private | lyn2 |
| 8 | 30651769 | 30651769 | G | A | PPP2CB | missense | p.P172L | FALSE | Private | lyn2 |
| 6 | 2896388 | 2896388 | C | T | SERPINB9 | missense | p.A69T | FALSE | Private | lyn2 |
| 2 | 174783430 | 174783430 | G | A | SP3 | missense | p.H572Y | FALSE | Private | lyn2 |
| 5 | 147443645 | 147443645 | C | T | SPINK5 | missense | p.A13V | FALSE | Private | lyn2 |
| X | 12905987 | 12905987 | G | A | TLR7 | missense | p.C787Y | FALSE | Private | lyn2 |
| 16 | 4922936 | 4922936 | G | A | UBN1 | splicing |  | FALSE | Private | lyn2 |
| 1 | 26612008 | 26612008 | C | T | UBXN11 | missense | p.G234S | FALSE | Private | lyn2 |
| 1 | 26611968 | 26611968 | C | T | UBXN11 | missense | p.G247E | FALSE | Private | lyn2 |
| 2 | 20189774 | 20189774 | C | T | WDR35 | missense | p.M1I | FALSE | Private | lyn2 |
| 2 | 71650878 | 71650878 | G | A | ZNF638 | missense | p.E1412K | FALSE | Private | lyn2 |
| 12 | 27089535 | 27089535 | C | T | INTS13 | missense | p.D68N | FALSE | Private | lyn2 |
| X | 41010293 | 41010293 | A | AGCGCCTCC | USP9X | frameshift ins | p.E582fs | FALSE | Private | lyn2 |
| 22 | 18907238 | 18907238 | C | T | PRODH | missense | p.R254Q | FALSE | Private | lyn3 |
| 2 | 166908477 | 166908477 | G | C | SCN1A | missense | p.A239G | FALSE | Private | lyn3 |
| 1 | 112303441 | 112303441 | G | T | DDX20 | missense | p.R265I | FALSE | Private | lyn3 |
| 12 | 13366720 | 13366720 | G | A | EMP1 | missense | p.R94Q | FALSE | Private | lyn3 |
| 1 | 213046056 | 213046056 | C | T | FLVCR1 | missense | p.A307V | FALSE | Private | lyn3 |
| 5 | 176637808 | 176637808 | C | A | NSD1 | missense | p.P534H | TRUE | Private | lyn3 |
| 16 | 23505659 | 23505659 | A | G | GGA2 | missense | p.S73P | FALSE | Private | BM |
| 1 | 185892590 | 185892590 | C | G | HMCN1 | missense | p.L364V | FALSE | Private | BM |
| 1 | 117504292 | 117504292 | T | C | PTGFRN | splicing |  | FALSE | Private | BM |
| 5 | 148892770 | 148892770 | T | G | CSNK1A1 | missense | p.L153F | FALSE | Private | BM |
| 6 | 30673836 | 30673836 | C | T | MDC1 | missense | p.V1042I | FALSE | Private | BM |
| 14 | 78082907 | 78082907 | C | T | SPTLC2 | missense | p.G6R | FALSE | Private | BM |
| 19 | 47258712 | 47258712 | G | A | FKRP | missense | p.R2Q | FALSE | Private | BM |
| 20 | 60581771 | 60581771 | G | C | TAF4 | missense | p.A673G | FALSE | Private | BM |
| X | 44918581 | 44918581 | G | A | KDM6A | nonsense | p.W355X | FALSE | Private | BM |
